# Ultrahigh Performance of Novel Capacitive Deionization Electrodes based on A Three-Dimensional Graphene Architecture with Nanopores

**DOI:** 10.1038/srep18966

**Published:** 2016-01-05

**Authors:** Wenhui Shi, Haibo Li, Xiehong Cao, Zhi Yi Leong, Jun Zhang, Tupei Chen, Hua Zhang, Hui Ying Yang

**Affiliations:** 1 Pillar of Engineering Product Development, Singapore University of Technology and Design, 8 Somapah Road, 487372, Singapore; 2 Center for Programmable Materials, School of Materials Science and Engineering, Nanyang Technological University, 50 Nanyang Avenue, 639798, Singapore; 3 College of Materials Science and Engineering, Zhejiang University of Technology, 18 Chaowang Road, Hangzhou 310014, China; 4 School of Electrical and Electronic Engineering, Nanyang Technological University, 50 Nanyang Avenue, 639798, Singapore

## Abstract

In order to achieve optimal desalination during capacitive deionization (CDI), CDI electrodes should possess high electrical conductivity, large surface area, good wettability to water, narrow pore size distribution and efficient pathways for ion and electron transportation. In this work, we fabricated a novel CDI electrode based on a three-dimensional graphene (3DG) architecture by constructing interconnected graphene sheets with in-plane nanopores (NP-3DG). As compared to 3DG, NP-3DG features a larger specific surface area of 445 m^2^ g^**−**1^ and therefore the higher specific capacitance. The ultrahigh electrosorptive capacity of NP-3DG predicted from Langmuir isotherm is 17.1 mg g^**−**1^ at a cell potential of 1.6 V. This can be attributed to the interconnected macropores within the graphene networks and nanopores on graphene sheets. Both of macropores and nanopores are favorable for enhancing CDI peroformance by buffering ions to reduce the diffusion distances from the external electrolyte to the interior surfaces and enlarging the surface area.

Water scarcity is a crippling issue of global proportions and based on a recent water development report from the United Nations, this problem will only worsen in the next 15 years or so. Among current technologies employed to combat the water crisis, desalination has emerged as a key strategy to solve worldwide water shortage^1^,^2^,^3^. Commercial desalination technologies include reverse osmosis (RO) and thermal processes. However these processes consume large amounts of energy and have high maintenance costs. On the contrary, capacitive deionization (CDI) is membrane free and operates at low voltages which make it a promising low cost water desalination technique^4^,^5^,^6^.

The concept of CDI follows the working principle of an electrical double-layer capacitor (EDLC). When an external voltage is applied, salt ions are electro-adsorbed on the electrical double-layer formed between the solution and the porous electrode interface (see Fig. 1). Once these pores are saturated with salt ions, a reverse voltage or a short circuit is applied to regenerate the electrodes. Therefore, the CDI performance depends strongly on physical properties and internal structure of the electrode materials. In principle, electrode materials for CDI should have high electrical conductivity, large surface areas, good wettability to water and a narrow pore size distribution^7^,^8^.

Generally, carbon materials with high electrical conductivity and tunable structural properties have been considered as promising electrode materials for CDI^9^,^10^,^11^. The list of carbon materials reported in literature includes graphene^12^, carbon nanotube^13^,^14^, activated carbon^15^,^16^, carbon aerogel^17^,^18^ and their composites^19^,^20^,^21^,^22^. Among these materials, the unique properties of high intrinsic electrical conductivity, remarkable mechanical properties and exceptionally high theoretical surface area of 2,630 m^2^ g^−1^ make graphene an ideal candidate for CDI application^23^,^24^,^25^. Due to the requirements on mass production and facile preparation, one of the most convenient ways is to synthesize graphene oxide (GO) and followed by reducing^26^. However, the reduction process can cause GO sheets to agglomerate due to their strong π–π interactions and this leads to uncontrollable pore size distribution and low accessible surface areas, which significantly limit their practical usage in CDI applications^27^,^28^. Consequently, it decreases the EDLC property of the electrodes and deteriorates the CDI performance.

To alleviate the issue of agglomeration, the mostly used method is to add “spacers” between the graphene sheets. Various “spacers” such as metal oxides, conductive polymers or carbon materials were chosen to incorporate into the interlayers of graphene^29^,^30^,^31^,^32^,^33^. Constructing three-dimensional (3D) graphene materials with macroporous structure is another effective approach to suppress the restacking of graphene^34^,^35^. For example, a 3D macroporous graphene architecture with wide pore size distribution was fabricated by using polystyrene microspheres as sacrificial templates^36^.

Recently, graphene sheets full of nanopores in their basal planes have been explored by various methods, such as laser scribing^37^, helium ion beam drilling^38^, and chemical etching^39^. Although existing studies have found potential applications of nanoporous graphene in fields such as energy storage devices and gas separation, the potential role of this material for water desalination remains largely unexplored^40^,^41^,^42^,^43^.

Herein, we proposed a novel CDI electrode based on a three-dimensional graphene (3DG) architecture, which is composed of both macropores and in-plane nanopores (NP-3DG). The as-prepared NP-3DG exhibits a significantly high specific surface area of 445 m^2^ g^−1^, as well as a favorable pore size distribution of approximately a few nanometers. To the best of our knowledge, there has not been any report on the fabrication of CDI electrodes based on graphene materials with above designed structures. It is believed that the interconnected macropores within graphene networks enhance desalination performance by buffering ions to shorten the diffusion distances from the external electrolyte to the interior surfaces. Furthermore, the nanopores on graphene sheets can further enlarge the surface area and hence, improve both electrosorption capacity and ion transport (see Fig. 1). As expected, an ultrahigh electrosorptive capacity of 17.1 mg g^−1^ was achieved at a cell potential of 1.6 V, which is among the best performance of previous reported graphene-based electrodes for CDI.

## Results and Discussion

**Figure 2:**
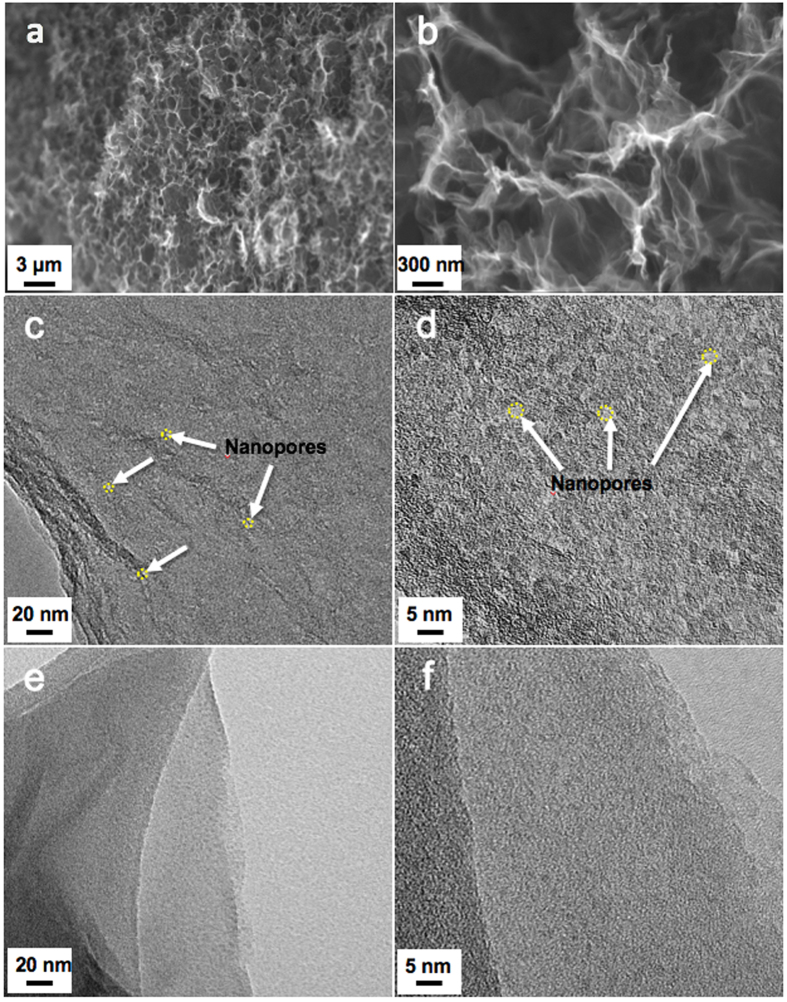
(**a**,**b**) SEM images of freeze-dried NP-3DG at different magnifications. TEM images of (**c**,**d**) NP-3DG and (**e**,**f**) 3DG. White arrows in (**c**,**d**) indicates nanopores on graphene sheets.

NP-3DG was prepared by a facile hydrothermal process, in which GO sheets were converted to reduced GO (rGO) and assembled into a three-dimensional architecture (See Materials Synthesis Section). During this process, nanopores were generated in the basal plane of graphene through a H_2_O_2_-induced chemical etching process, where carbon atoms of graphene were etched with H_2_O_2_ and gradually extended into nanopores^44^,^45^. Figure 2a,b show the morphology of freeze-dried NP-3DG. The rGO sheets are interconnected to form a highly porous 3D network with well-defined pore sizes ranging from sub-micrometers to several micrometers. The high-magnification scanning electron microscopy (SEM) image in Fig. 2b clearly indicates that the obtained 3D architecture is composed of few-layered graphene sheets. Furthermore, transmission electron microscopy (TEM) studies confirm the presence of abundant in-plane pores with sizes of a few nanometers that are distributed over whole graphene sheets (see Fig. 2c,d). In a control experiment, 3DG was prepared without the addition of H_2_O_2_ and is shown in Fig. 2e,f which demonstrates a smooth graphene morphology without presenting nanopores. Hence, it is expected that the interconnected macroporous materials are favor to improve the specific surface area and optimize the porous structure, which are very desirable for high-performance CDI.

**Figure 3:**
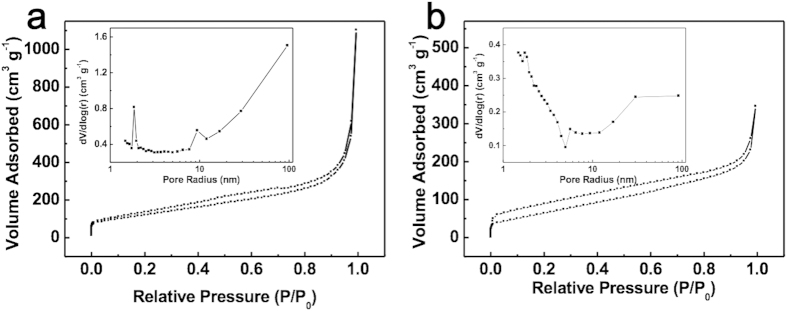
Nitrogen sorption isotherms of (**a**) NP-3DG and (**b**) 3DG. Insets are the pore size distribution of NP-3DG and 3DG, respectively.

To further investigate the porous texture of NP-3DG, N_2_ adsorption/desorption test was performed. As shown in Fig. 3a,b, NP-3DG has a specific surface area of 445 m^2^ g^−1^, which is significantly higher than that of 3DG (247 m^2^ g^−1^). This enhancement in surface area can be attributed to the existence of abundant in-plane nanopores. The pore size distribution profiles of NP-3DG and 3DG (insets of Fig. 3a,b) further indicate NP-3DG possesses a narrower size distribution of around 4 nm which is consistent with TEM observations. In the CDI process, when the width of a pore is smaller than a specific value (cutoff pore width), it does not contribute to the total electrosorption capacity due to an overlap of electrical double-layers. This effect is evident in microporous 3DG electrodes. The freeze drying process used to prepare Brunauer–Emmett–Teller (BET) sample may induce partial restacking of some graphene layers and thus reduce the specific surface area, hence we have also used the Methylene Blue (MB) adsorption method to more accurately determine the solvated surface area. Using this method, we found the specific surface area of NP-3DG to be 1,060 m^2^ g^−1^ and 3DG to be 730 m^2^ g^−1^.

Raman spectroscopy was used to characterize the structure of NP-3DG (see Fig. S1 in SI). The D-band of graphitic materials is related to the density of disorder sites that are due to the breathing mode of k-point phonons of A_1g_ symmetry while the G-band is associated with the conjugated structure of sp^2^ carbon domains^46^,^47^. The intensity ratio of the D band to G band (I_D_/I_G_) for NP-3DG is about 1.1, whereas the value for 3DG is 1.05. The slightly higher D/G ratio of NP-3DG may be attributed to additional defects around the nanopores in NP-3DG.

**Figure 4:**
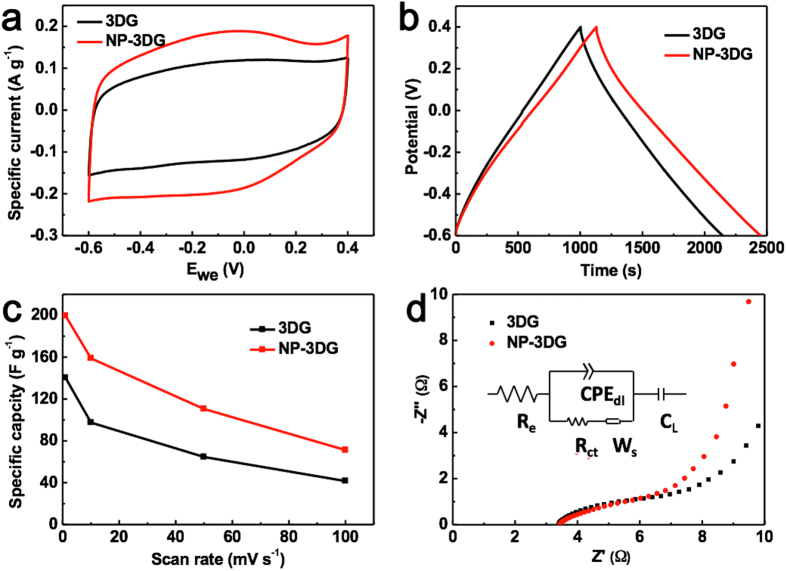
(**a**) CV curves of NP-3DG and 3DG measured at a scan rate of 1 mV s^−^^1^ (**b**) Charge/discharge curves of NP-3DG and 3DG at a current density of 0.1 A g^−^^1^ (**c**) Specific capacity of NP-3DG and 3DG at various scan rates (**d**) Nyquist plots of NP-3DG and 3DG electrodes and equivalent circuit (inset) at 1 M NaCl aqueous solution.

The electrochemical behavior of the electrodes were examined by Cyclic voltammetry (CV) measurements. Figure 4a shows the CV curves of NP-3DG and 3DG at a scan rate of 1 mV s^−1^ in 1 M NaCl solution with a potential window from −0.6 to 0.4 V, where no obvious redox peak is observed from the CV curves. This suggests a typical EDLC behavior of both electrodes, which is due to the Coulombic interactions, rather than the electrochemical reduction/oxidation reactions^48^,^49^. The charge/discharge curves of NP-3DG and 3DG (Fig. 4b) show typical symmetric shape in accordance with CV curves. The specific capacitances were calculated from the CV curve at various scan rates and shown in Fig. 4c. Regardless of the scan rate, NP-3DG has a much higher specific capacitance as compared to that of 3-DG. This is ascribed to its higher specific surface area and pore volume. Electrochemical impendence spectra (EIS) analysis has been recognized as one of the principal methods to examine the inner resistivity of a carbon electrode. The Nyquist profiles of NP-3DG and 3DG electrodes in 1 M NaCl aqueous solution are presented in Fig. 4d The plots of both electrodes display similar shapes, consisting of a linear trait at the low frequency region and a small quasi-semicircle at the high frequency range. The point intersecting the real axis is related to the equivalent series resistance (R_e_) of the electrode, which is a result of the ionic resistance of salty water, the intrinsic resistance of electrodes, and the contact resistance at the interface of active material/current collectors. It is found that the R_e_ of the NP-3DG electrode is lower than that of the 3DG electrode. In the low frequency region, the inclined line is derived from the typical EDLC. The inclined line of the NP-3DG electrode is more vertical than that of the 3DG, suggesting the NP-3DG electrode displays a more ideal capacitive behavior, because salty ions diffuse faster and more easily into the 3D interconnected macroporous structure with abundant nanopores.

**Figure 5:**
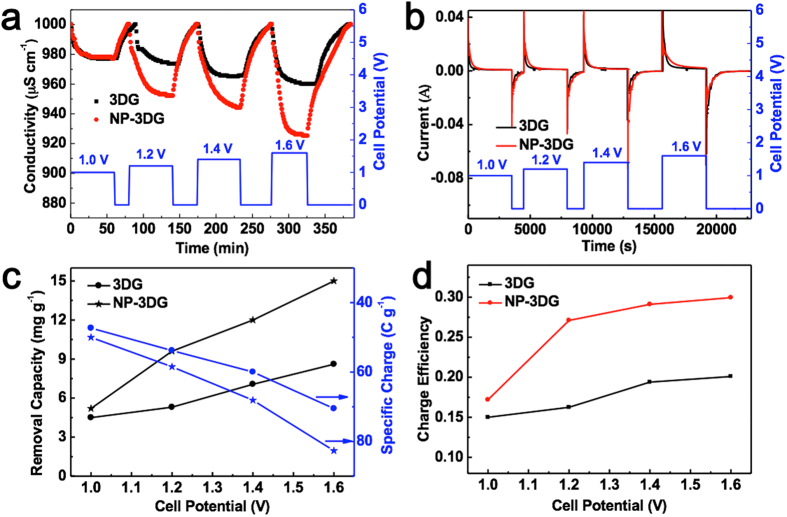
(**a**) The electrosorption-desorption performance and (**b**) the corresponding current response of NP-3DG and 3DG electrodes in NaCl solution with a intial concentration of 500 mg L^−1^ by varying the cell voltage from 1.0 to 1.6 V (**c**) Removal capacity and specific charge of NP-3DG and 3DG electrodes with respect to the cell potential (**d**) Charge efficiency of NP-3DG and 3DG electrodes at each cell potential.

To determine the electrosorption performance of NP-3DG and 3DG electrodes, batch mode CDI experiments were carried out in NaCl solution with an initial concentration of 500 mg L^−1^. In a typical electrosorption-desorption cycle, a certain cell voltage was applied across the electrodes for a period of time before it was shorted. The corresponding current response for electrodes and the conductivity of NaCl solution were recorded simultaneously and independently. The CDI performance can be examined from the conductivity variation of NaCl solution during the charging process. Figure 5a,b represent the typical electrosorption-desorption cycle and the corresponding current response for NP-3DG and 3DG electrodes under a cell voltage ranging from 1.0 to 1.6 V. As shown in Fig. 5a, once an electric field was applied, the solution conductivity began to decrease sharply, which indicates the adsorption of salt ions. Then, the change of solution conductivity gradually became smaller until equilibrium was reached. Subsequently, the cell was short-circuited, and the conductivity rapidly returned to the initial value due to the desorption of ions. Moreover, with the increase of applied voltage, more ions are adsorbed and a greater decrease in conductivity is observed. Obviously, the descending rate of conductivity for NP-3DG electrode is faster than that of 3DG, indicating that salty ions are more favorable to adsorbing on NP-3DG. Figure 5b is the corresponding transient current curve, which shows similar changes with that of solution conductivity, confirming the drop of conductivity comes from electrosorption.

In addition, conductivity variations are reproducible for several cycles of electrosorption and desorption, indicating good regeneration of electrodes. The salt removal capacity (mg g^−1^) is used to determine the performance of a CDI electrode, which is often defined as: 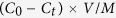, where C_0_ and C_t_ are initial and final concentration (mg L^−1^), respectively, V is the volume of solution and M is the total mass of the electrode. Figure 5c compares the salt removal capacities of NP-3DG and 3DG electrodes in NaCl solutions under different concentrations. With an increase of cell voltage, removal capacities of both NP-3DG and 3DG increase. Under constant voltage operation, the electrosorption capacity increases with increasing NaCl concentrations, which is due to the enhanced mass transfer rate of ions inside the pores and reduced overlapping effects by a higher concentration of solution. Significantly, NP-3DG has a much higher salt removal capacity (15 mg g^−1^) than that of 3DG (8.3 mg g^−1^) at a cell potential of 1.6 V. Charge efficiency (Λ) is another functional tool used to gain insight into the double layer formed at the interface between the electrode and solution, as calculated according to the following equation: 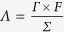, where F is the Faraday constant (96485 C mol^−1^), Γ is the electrosorption capacity (mol g^−1^) and Σ (charge, C g^−1^) is obtained by integrating the corresponding current. According to above equation, the charge efficiencies of NP-3DG and 3DG are plotted versus cell potential (see Fig. 5d). The higher charge efficiency of NP-3DG may be due to its 3D structure with hierarchical pores which facilitates the ion diffusion and charge transfer.

**Figure 6:**
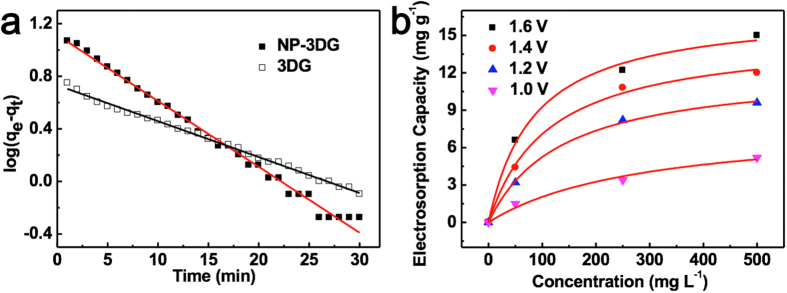
(**a**) The electrosorption kinetics of NP-3DG and 3DG electrodes in NaCl solution with a intial concentration of 500 mg L^−1^ at a cell voltage of 1.4 V (**b**) The electrosorption isotherm of NP-3DG electrode at cell potentials of 1.0, 1.2, 1.4 and 1.6 V, respectively.

Adsorption kinetics, which reflect the adsorption rate, is an important characteristic of adsorbents. It can be determined by using Lagergren’s pseudo-first-order adsorption kinetics, which is often presented as: 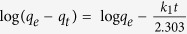, where q_e_ (mg g^−1^) and q_t_ (mg g^−1^) are the amount of NaCl adsorbed at equilibrium and time t (min), respectively. k_1_ (mg g^−1^ min^−1^) is the adsorption rate constants of pseuduo-first-order equations. Figure 6a shows the linear fit between the experimental data and the above equation. The regression coefficients for NP-3DG and 3DG are 0.9924 and 0.9958, respectively, indicating good simulations of both electrodes since they are very close to 1. The rate constants calculated from the slope of the fitting lines are 0.1172 and 0.0629 for NP-3DG and 3DG electrode, respectively. The high-rate constant for NP-3DG is ascribed to the quick access of ions within the electrode. It should be noted that efficient electrosorption and desorption is desirable for practical CDI devices to ensure maximum desalination of salty water.

Further experiments were carried out in NaCl solutions with initial concentrations ranging from 50 mg L^−1^ to 500 mg L^−1^ to investigate the electrosorption isotherms of the electrodes. The adsorption isotherm is useful to evaluate the adsorption capacity of the electrode material and understand the inherent adsorption mechanism. Figure 6b shows the electrosorption isotherms of NP-3DG at cell potentials of 1.0, 1.2, 1.4 and 1.6 V, respectively. The Langmuir isotherm was used to fit the experimental data: 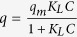, where C is the equilibrium concentration (mg L^−1^), q is the amount of adsorbed NaCl (mg g^−1^), and q_m_ is the maximum adsorption capacity corresponding to complete monolayer coverage (mg g^−1^). Table 1 shows the determined parameters and regression coefficient q_m_, K_L_ and r_L_^2^ of Langmuir isotherm for NP-3DG electrode. The q_m_ value of the NP-3DG electrode as calculated by the Langmuir equation is much higher than that of the 3DG electrode (see Fig. S2 and Table S1 for details of the 3DG electrode), implying enhanced electrosorption performance of the NP-3DG electrode.

Compared with the reported graphene-based electrode materials, our result shows a comparable and even higher performance in the terms of electrosorption capacity (see Table S2). The excellent CDI performance of NP-3DG is attributed to the following reasons: 1) Constructing a 3D graphene architecture with macroporous structure is an effective approach to suppress the restacking of graphene and preserve the high surface area of graphene. 2) The interconnected macropores within graphene networks are favorable for buffering ions to shorten the diffusion distances from the external electrolyte to the interior surfaces, and the nanopores in thin walls can enhance the ion transport and electrosorption capacity.

## Conclusion

In conclusion, we have demonstrated a new CDI electrode design by creating a highly interconnected 3D graphene architecture with good electrical conductivity and hierarchical porosity, which can ensure efficient electron and ion transport. An ultrahigh electrosorptive capacity of 17.1 mg g^−1^ at a cell potential of 1.6 V was obtained with NP-3DG-based CDI electrodes.

## Methods

### Preparation of GO

GO was prepared by a modified Hummers method reported in our previous works^50^. In brief, 0.3 g of graphite was added into a mixture of 2.4 mL of 98% H_2_SO_4_, 0.5 g of K_2_S_2_O_8_, and 0.5 g of P_2_O_5_, and the solution was kept at 80 °C for 4.5 h. The resulting preoxidized product was cleaned by water and dried. After the preoxidized product was added into 12 mL of 98% H_2_SO_4_, followed by slow addition of 1.5 g of KMnO_4_ with the temperature kept at <20 °C in order to avoid overheating and explosion, the solution temperature was increased to 35 °C and maintained for 2 h. Then, 25 mL of H_2_O was added. After 2 h, an additional 70 mL of H_2_O was added to dilute the solution, and 2 mL of 30% H_2_O_2_ was injected into the solution to completely react with the excess KMnO_4_. A bright yellow solution was obtained. Then, the resulting mixture was washed with HCl aqueous solution (1:10 in volume) and H_2_O, and the graphite oxide was obtained. The obtained graphite oxide was dispersed in water with a certain concentration and subsequently sonicated to obtain GOs.

### Preparation of NP-3DG and 3DG

NP-3DG was prepared by a modified method based on Duan *et al.’*s work^45^. Typically, 200 μl H_2_O_2_ solution (30% H_2_O_2_) was added into 50 ml 2 mg ml^−1^ GO aqueous dispersion in a 120 ml Teflon lined autoclave. The mixture was sealed and heated at 180 °C for 12 h and naturally cooled down to room temperature and the as-prepared NP-3DG was taken out with a pair of tweezers and immersed in pure water to remove any impurities for the following experiments. 3DG was prepared using the same procedure without adding H_2_O_2_.

### Characterization

The morphology of the samples were carried out by field-emission scanning electron microscopy (FE-SEM, JEOL JSM-7600F) and transmission electron microscopy (TEM, JEM-2010). Nitrogen adsorption/desorption was measured using an automated gas sorption analyzer (Autosorb-iQ, Quantachrome Instruments, USA). The specific surface area (SSA) was calculated from N_2_ adsorption data by the multipoint Brunauer–Emmett–Teller (BET) method. The pore size distribution was determined using the Barrett-Joyner-Halenda (BJH) method. Methylene Blue (MB) dye adsorption method was employed to measure the specific surface areas of NP-3DG and 3DG. MB adsorption is a standard method for measuring the specific surface area of graphitic materials, with 1 mg of adsorbed MB molecules covering 2.54 m^2^ of surface area^51^. The surface areas were calculated by adding a piece of NP-3DG or 3DG into a standard concentration of MB in DI water for a total of 24 h to reach adsorption equilibrium. The MB concentration was determined by analyzing the supernatant through UV–vis spectroscopy (PerkinElmer Lambda 750 S UV–Vis spectrophotometer) at a wavelength of 665 nm and compared with the initial standard concentration of MB before interacting with NP-3DG or 3DG. Raman spectroscopy was obtained by a confocal Raman system with the 532 nm laser excitation (WITec Instruments Corp Gernmany). Cyclic voltammetry (CV) and electrochemical impendence spectra (EIS) measurements were carried out in 1 M NaCl solution by using electrochemical workstation (VMP3, Bio-logic, France) in a three-electrode mode, including a standard calomel electrode as reference electrode and a platinum foil as counter electrode.

### Fabrication of NP-3DG and 3DG electrodes

Slices of NP-3DG with a thickness of ~2 mm were first cut from the as-prepared cylindrical NP-3DG. Subsequently, the NP-3DG slices were placed on the graphite electrode and compressed using hydraulic press during which the squeezed water was removed by filter papers. The samples were kept under a 150-MPa pressure for 1 min to form ~14 μm well-adhered films on the graphite electrode. The size of the electrode was 60 mm × 60 mm, two NP-3DG films (both with a net-weight of ~60 mg) on separate graphite papers were directly used as electrodes without any other additives or further treatments such as drying and thermal annealing. The fabrication of 3DG electrode is same with NP-3DG.

### Electrosorption test

Batch-mode experiments were conducted in a continuously recycling system including a CDI unit cell, conductivity monitor and current recorder. During each experiment, the solution was continuously pumped by a peristaltic pump into a unit cell and the effluent returned to the feed tank. In a typical experiment, the aqueous solution was prepared using pure sodium chloride (NaCl) with an initial concentration of 500 mg L^−1^, corresponding to the conductivity of 1000 μS cm^−1^. The volume and the temperature of the solution were maintained at 50 mL and 298 K, respectively. The relationship between conductivity and concentration was obtained according to a calibration table made prior to the experiment. A direct voltage of 1.0 V to 1.6 V with an interval of 0.2 V was applied on the CDI unit cell. The variations in conductivity and current were recorded simultaneously and independently. It should be noted that hydrolysis of water didn’t take place when the applied voltage is higher than 1.2 V due to the existence of resistance in the whole circuit and over potential between two porous electrode.

## Additional Information

**How to cite this article**: Shi, W. *et al.* Ultrahigh Performance of Novel Capacitive Deionization Electrodes based on A Three-Dimensional Graphene Architecture with Nanopores. *Sci. Rep.* **6**, 18966; doi: 10.1038/srep18966 (2016).

## Supplementary Material

Supplementary Information

## Figures and Tables

**Figure 1:**
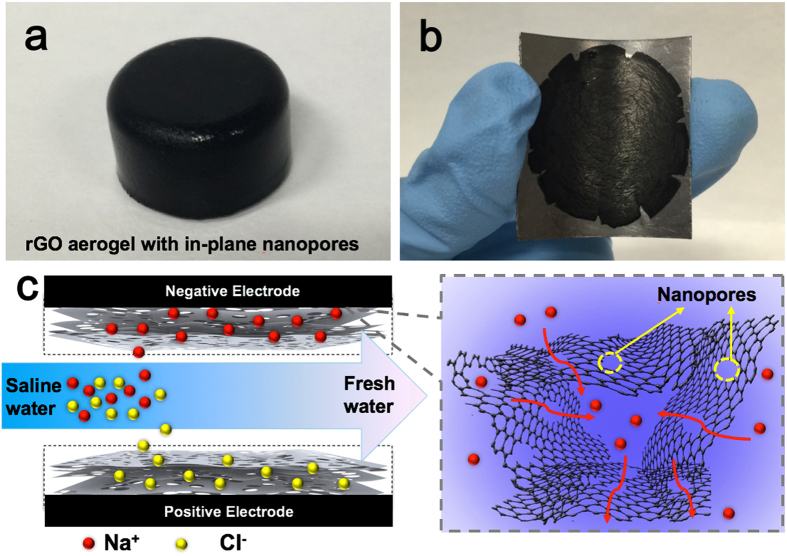
Photographs of (**a**) as-prepared rGO hydrogel with in-plane nanopores (NP-3DG) and (**b**) CDI electrode, (**c**) Schematic diagram of the CDI process.

**Table 1:** Parameters determined from Langmuir isotherm of NP-3DG electrode.

Potential (V)	q_m_ (mg g^−1^)	KL	rL^2^
1.0	8.01	0.0034	0.9789
1.2	12.36	0.0074	0.9983
1.4	15.02	0.0089	0.9951
1.6	17.09	0.0118	0.9939
