# Supplementary material for: Ultrahigh Performance of Novel Capacitive Deionization Electrodes based on A Three-Dimensional Graphene Architecture with Nanopores
Source: Sci Rep. 2016 Jan 5;6:18966. doi: 10.1038/srep18966 (PMC4700447; doi:10.1038/srep18966)
Supplement: Supplementary Information [file srep18966-s1.pdf]

Supporting Information

SUBJECT AREAS

ELECTROCHEMISTRY GRAPHENE

Correspondence and requests for materials should be addressed to H.Y. (yanghuiying@sutd.edu.sg)

Ultrahigh Performance of Novel Capacitive Deionization Electrodes based on A Three-Dimensional Graphene Architecture with Nanopores

Wenhui Shi[1], Haibo Li[1], Xiehong Cao[23], Zhi Yi Leong[1], Jun Zhang[14], Tupei Chen[4], Hua Zhang[2], and Hui Ying Yang[1,*]

[1] Pillar of Engineering Product Development, Singapore University of Technology and Design, 8 Somapah Road, 487372, Singapore.

[2] Center for Programmable Materials, School of Materials Science and Engineering, Nanyang Technological University, 50 Nanyang Avenue, Singapore 639798, Singapore.

[3] College of Materials Science and Engineering, Zhejiang University of Technology, 18 Chaowang Road, Hangzhou 310014, China.

[4] School of Electrical and Electronic Engineering, Nanyang Technological University, 50 Nanyang Avenue, 639798, Singapore.

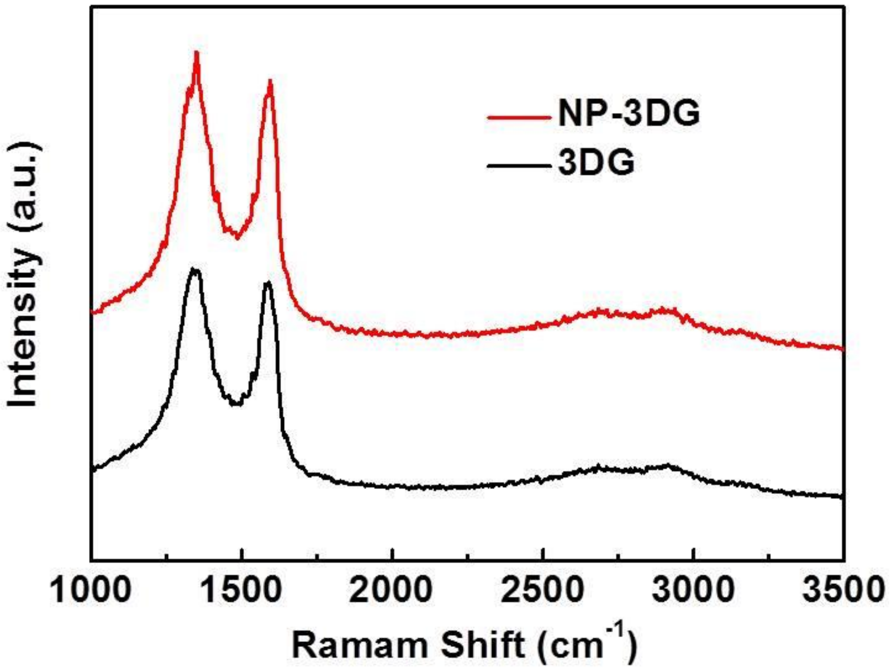

Figure S1. Raman Spectra of NP-3DG and 3DG.

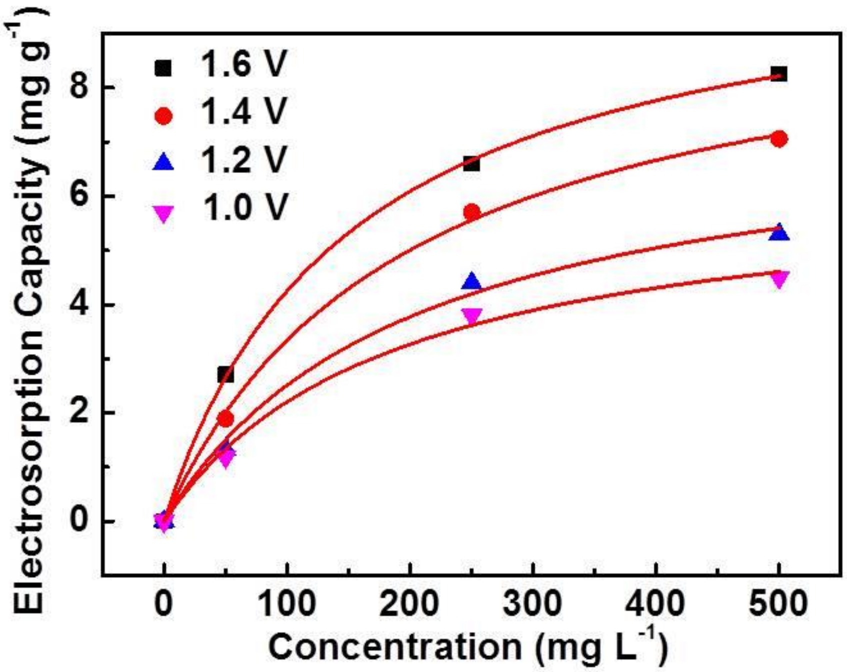

Figure S2. The electrosorption isotherm of 3DG electrode at cell potentials of 1.0, 1.2, 1.4 and 1.6 V, respectively.

Table S1. Parameters determined from Langmuir isotherm of 3DG electrode.

**Potential (V)**	**q_m (mg g^{-1})**	**K_L**	**r_L^2**
1.0	6.32	0.0053	0.9927
1.2	7.60	0.0049	0.9933
1.4	9.96	0.0051	0.9982
1.6	10.72	0.0066	0.9997

Table S2. Comparison of electrosorption capacities of various graphene-based CDI electrodes.

Electrode Material	Cell Voltage (V)	Initial NaCl Concentration (mg L^{-1})	Electrosorption Capacity (mg g^{-1})	Specific Surface Area (m^2 g^{-1})
**CNT/graphene composite**[1]	1.2	500	1.4	438.6
**AC/graphene composite**[2]	1.2	500	2.94	779
**Graphene aerogel**[3]	1.2	500	9.9	-
**3D-macroporous graphene architecture**[4]	1.6	50	3.9	305
**Sponge-templated graphene**[5]	1.5	53	4.95	305
**Graphene sponge**[6]	1.5	50	5.52	356
**NP-3DG (this work)**	1.6	500	17.09	445
**NP-3DG (this work)**	1.6	500	10.72	247

Reference

1 Li, H., Liang, S., Li, J. & He, L. The capacitive deionization behaviour of a carbon nanotube and reduced graphene oxide composite. *J. Mater. Chem.* **1**, 6335-6341, (2013).

2 Li, H., Pan, L., Nie, C., Liu, Y. & Sun, Z. Reduced graphene oxide and activated carbon composites for capacitive deionization. *J. Mater. Chem.* **22**, 15556-15561, (2012).

3 Yin, H. *et al.* Three-Dimensional Graphene/Metal Oxide Nanoparticle Hybrids for High-Performance Capacitive Deionization of Saline Water. *Adv. Mater.* **25**, 6270-6276, (2013).

4 Wang, H. *et al.* Three-dimensional macroporous graphene architectures as high performance electrodes for capacitive deionization. *J. Mater. Chem.* **1**, 11778-11789, (2013).

5 Yang, Z.-Y. *et al.* Sponge-Templated Preparation of High Surface Area Graphene with Ultrahigh Capacitive Deionization Performance. *Adv. Funct. Mater.* **24**, 3917-3925, (2014).

6 Xu, X. *et al.* Facile synthesis of novel graphene sponge for high performance capacitive deionization. *Sci. Rep.* **5**, (2015).
